# *Helicobacter canis* bacteraemia in a rheumatoid arthritis patient treated with tofacitinib: case report and literature review

**DOI:** 10.1186/s12941-021-00426-x

**Published:** 2021-04-08

**Authors:** Matic Mihevc, Metka Koren Krajnc, Maja Bombek Ihan, Iztok Holc

**Affiliations:** 1 Division of Internal Medicine, Department of Rheumatology, University Medical Centre Maribor, Ljubljanska ulica 5, 2000 Maribor, Slovenia; 2 National Laboratory for Health, Environment and Food, Maribor, Slovenia; 3 Faculty of Medicine, Department of Internal Medicine, University of Maribor, Maribor, Slovenia

**Keywords:** *Helicobacter canis*, Zoonosis, Rheumatoid arthritis, Immunocompromised, Tofacitinib, Hypogammaglobulinemia, Treatment

## Abstract

**Background:**

Non-*Helicobacter pylori* species (NHPS) are newly emerging bacteria that naturally inhabit birds and mammals apart from humans and rarely cause diseases in humans. In recent years, a rise in the number of cases associated with NHPS infections in humans has been observed. Among them, infections with *Helicobacter (H.) canis* are sporadic and challenging to recognise clinically. To date, ten cases of *H. canis* infections in mainly immunocompromised humans have been reported in the literature. Transmission pathway is most likely zoonotic via the faecal-oral route during close contacts with dogs and cats or may result from a contaminated sheep milk intake. No clear guidelines for successful antibiotic regimen are known. Important additional risk factor for infection might be biologic agents and Janus kinase inhibitors (JAKi) used in the treatment of rheumatoid arthritis (RA) and other conditions. Herein we present the first case of *H. canis* bacteraemia in a RA patient treated with novel JAKi tofacitinib.

**Case presentation:**

A 65-year-old female patient with RA and rituximab-induced hypogammaglobulinemia treated with tofacitinib, methotrexate, and methylprednisolone came to a planned visit in our outpatient rheumatology clinic. She presented with a history of back pain that significantly worsened 2 days before visit. She had numbness and tingling sensation in both legs and muscle weakness. Neurological examination was within a normal range. The patient was afebrile, had no chills, and was haemodynamically stable. She was in close contact with her pet dogs. Laboratory examination showed increased markers of inflammation. She was found to have *H. canis* bacteraemia with underlying multilevel degenerative lumbar spinal stenosis. Identification of *H. canis* was performed by MALDI-TOF MS and 16 S rRNA gene sequence analysis of isolate from subcultured positive aerobic blood culture bottles. Antimicrobial susceptibility testing showed low minimum inhibitory concentrations to amoxicillin-clavulanate, cefotaxime, ceftriaxone, meropenem, and gentamicin. She was treated with combined antibiotic regimen (ceftriaxone, doxycycline) for 14 days, which resulted in total remission of the infection.

**Conclusions:**

Clinicians should recognise *H. canis* infection risk in patients with recent pet exposure and predisposing factors such as immunodeficiency disorders or diseases that demand immunosuppressive drug therapy. A minimum of two weeks of antibiotic therapy is suggested.

## Background

Helicobacter species are newly emerging Gram-negative spiral bacteria with unclear epidemiology and zoonotic transmission pathway [1]. To date, more than 35 species have been validated [2]. Generally, we classify them as *Helicobacter (H.) pylori* and non-*Helicobacter pylori* species (NHPS). *H. pylori* infects up to 50% of the human population and has been associated with peptic ulcers, chronic gastritis, and gastric cancer in humans [1]. Contrary, NHPS naturally inhabit birds and mammals apart from humans and rarely cause diseases in humans [1]. They have been associated with three groups of diseases: (a) gastrointestinal diseases (diarrhoea, gastric cancer), (b) bacteraemia, arthritis, cellulitis, and (c) hepatobiliary diseases (cholecystitis, hepatitis, hepatic cancer) [2–4]. In recent years, a rise in the number of NHPS infections in humans has been observed [1, 3, 4].

*Helicobacter canis* is a member of the NHPS that inhabits lower intestinal as well as hepatobiliary tract of dogs and cats [5]. According to an extensive PubMed/MEDLINE [6] search (keywords: *Helicobacter canis*, clinical case, infection) in April 2020, ten clinical cases of *H. canis* infection associated with human diseases have been described [5, 7–15]. An overview of clinical cases is presented in Table 1. Eight of them describe bacteraemia in patients with recent exposure to dogs or cats [5, 8–11, 13–15]. Lately, transmission between sheep and human contact was demonstrated, recognising sheep as an additional reservoir of *H. canis* [16]. Infection of humans with *H. canis* is most likely zoonotic via the faecal-oral route during close contacts with dogs and cats or may result from a contaminated sheep milk intake [14, 16]. Another predisposing factor might be an incompetent immune system of the host. Two previous cases described *H. canis* bacteraemia in patients with primary immunodeficiency diseases (X-linked hypogammaglobulinemia, common variable immune deficiency) [8, 13]. Another two cases described *H. canis* bacteraemia in patients treated with immunosuppressive drugs [11, 14].

**Table 1:** Review of *H. canis* human infections case reports

Author [reference]	Gender, age	Immune system competency	Pet contact	Clinical presentation	Detection	Treatment
Burnens et al. [7]	Male, 5 years	NR	NR	Gastroenteritis	Stool: Rotavirus, *H. canis*	NR
Gerrard et al. [8]	Male, 27 years	X-linked hypogammaglobulinemia	Dog	Recurrent fever, bilateral cellulitis	Blood culture: *H. canis, F. rapinni*	Doxycycline and metronidazole p.o. 5 months
Leemann et al. [9]	Male, 44 years	Immunocompetent	Dog, cats	Multifocal cellulitis, subfebrile	Blood culture: *H. canis*	Ceftriaxone 2 g daily i.v. 2 weeks
Prag et al. [10]	Female, 7 months	Immunocompetent	Cat	Acrocyanosis, intermittent fever	Blood culture: *H. canis*	Ampicillin and gentamicin i.v. followed by mecillinam p.o. 10 days
Alon et al. [11]	Male, 78 years	Diffuse large B cell lymphoma (R-CHOP)	Dogs	Subfebrile episodes, paroxysmal AF	Blood culture: *H. canis*	*H. pylori* eradication regimen
Tankovic et al. [12]	Female, 52 years	Crohn’s disease (mesalazine)	Cat	Epigastralgia	Duodenal biopsy: *H. canis*	Not treated
Abidi et al. [13]	Female, 57 years	CVID, pulmonary sarcoidosis (prednisone), splenectomy	Dogs, cats	Recurrent fever, chills, and sweats for 3 months	Blood culture: *H. canis*	Ceftriaxone 2 g daily i.v. 2 weeks, doxycycline 100 mg p.o. 6 weeks
Van der Vusse et al. [14]	Female, 41 years	Kidney transplant (tacrolimus, prednisone, mycophenolate mofetil)	Dog	Fever and cough	Blood culture: *H. canis*	Cefuroxime 1.5 g 3 times daily i.v. 3 days followed by ciprofloxacin 500 mg p.o. 10 days
Shakir et al. [5]	Male, 49 years	Immunocompetent	Dogs, cats	Cellulitis	Blood culture: *H. canis*	One dose of vancomycin 1.5 g i.v., doxycycline 100 mg twice daily p.o. 5 days, amoxicillin/clavulanic acid 500 mg daily p.o. 8 weeks
Gutiérrez-Arroyo et al. [15]	Male, 2 months	NR	NR	Cardiorespiratory arrest	Blood culture: *H. canis*	Death

*H Helicobacter*; *F Flexispira*; *NR* not reported; *p.o.* per os; *i.v.* intravenous; *R-CHOP* rituximab-cyclophosphamide, hydroxydaunorubicin hydrochloride (doxorubicin), oncovin (vincristine), prednisone; *AF* atrial fibrillation; *CVID* common variable immunodeficiency

One class of drugs that could represent a risk factor for *H. canis* infection and that interfere with host immune system are biologic agents and Janus kinase inhibitors (JAKi) used in the treatment of rheumatoid arthritis (RA) and other conditions [17]. Kourbeti et al. have shown that biologic agents used in the treatment of RA are associated with 1.5-2-fold increased risk of opportunistic infections, especially for mycobacterial and viral infections [18]. However, no data for NHPS are available.

One of the common biologic agents used for RA management is rituximab (RTX), a B-cell depleting drug [17]. A common side effect of RTX treatment is secondary hypogammaglobulinemia which has been observed in up to 40% of patients treated with RTX [19]. It is unclear whether a reduction in immunoglobulins correlates to a higher risk of infection. Recent large cohort study, investigating RTX infection profiles, concluded that only patients with lower immunoglobulin G (IgG) levels before RTX initiation had increased risk for severe infection events within 12 months of RTX initiation [20].

On the other hand, JAKi (e.g., tofacitinib, baricitinib) are novel drugs that reversibly inhibit JAK and interfere with cytokine function and signal transduction to the nucleus via the JAK/STAT pathway [17]. Compared to patients treated with biologic agents, patients treated with JAKi have a higher risk of herpes zoster infection, whereas the risk for other infections is like one observed in patients treated with biologic agents [21].

Herein we present a case of bacteraemia caused by *H. canis* in a female RA patient treated with tofacitinib and secondary rituximab-induced IgM/IgG hypogammaglobulinemia who was in close contact with her pet dogs. To the best of our knowledge, *H. canis* infection in a patient treated with tofacitinib has not been described before.

## Case presentation

A 65-year patient with seropositive RA and arterial hypertension came to a planned visit in our outpatient rheumatology clinic. She was diagnosed with RA in 1996 based on the American Rheumatism Association revised criteria for RA classification [22]. During 1996–2008, she was treated with methylprednisolone, a combination of methylprednisolone and methotrexate (MTX), and a combination of adalimumab, MTX and methylprednisolone, respectively. Between 2009 and 2018, she was treated with a combination of RTX, MTX, and methylprednisolone. However, in 2017 she noticed occasional itchy erythema on hands after RTX application. Additionally, the same year, low levels of IgM (< 0.18 g/L; reference value 0.4–2.3 g/L) and IgG (6.43 g/l; reference value 7.0–16.0 g/L) immunoglobulins were found. Therefore, in 2018, her treatment was changed to a combination of tofacitinib 5 mg twice daily, MTX 10 mg once weekly, and methylprednisolone 2 mg every second day.

In the outpatient clinic, the patient presented with a history of back pain that significantly worsened 2 days before visit with severe pain radiating to both legs, numbness and tingling sensation in both legs, and muscle weakness. She was unstable while walking. Neurological examination was within a normal range. The patient was afebrile, had no chills, and was haemodynamically stable. Laboratory examination showed normal blood leukocyte count (9.21 × 10^9^/L; reference value 4.00–10.00 × 10^9^/L), mild neutrophilia (7.88 × 10^9^/L; reference value 1.50–7.40 × 10^9^/L), mild anaemia (haemoglobin 116 g/l; reference value 120–150 g/L), increased C-reactive protein (98 mg/L; reference value < 5 mg/L), increased sedimentation rate (39 mm/h; reference value < 15 mm/h), and negative procalcitonin (0.07 ng/mL; reference value < 0.5 ng/mL). She was admitted to the hospital because of a suspected spondylodiscitis. Immunosuppressive therapy was discontinued. As the patient was afebrile, had no chills, no left shift leukocytosis, negative procalcitonin and there was a possibility that increased markers of inflammation reflected the baseline disease (RA), no empirical antibiotic therapy was initiated.

**Fig. 1:**
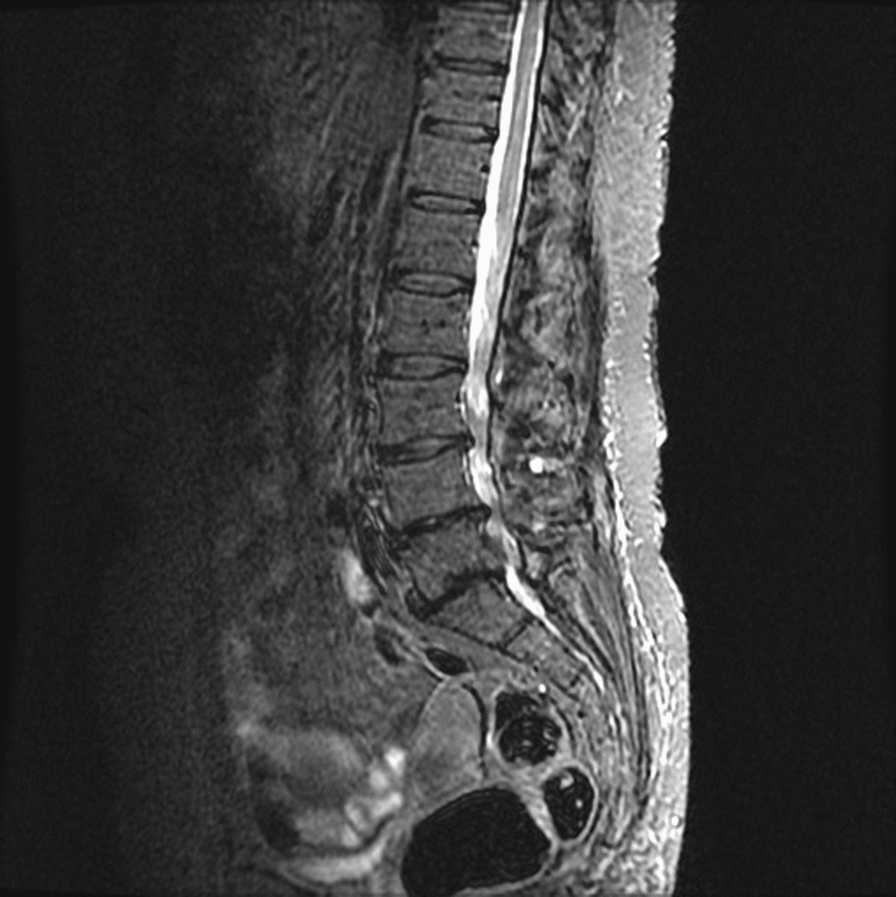
Magnetic resonance imaging of the lumbosacral spine revealing multilevel (L3-S1) degenerative lumbar spinal stenosis

The first day, a chest X-ray showed no abnormalities. Magnetic resonance imaging of the lumbosacral spine revealed multilevel (L3-S1) degenerative lumbar spinal stenosis (Fig. 1). However, no signs of spondylodiscitis were found. After consultation with a neurosurgeon, there was no indication for surgical intervention.

**Fig. 2:**
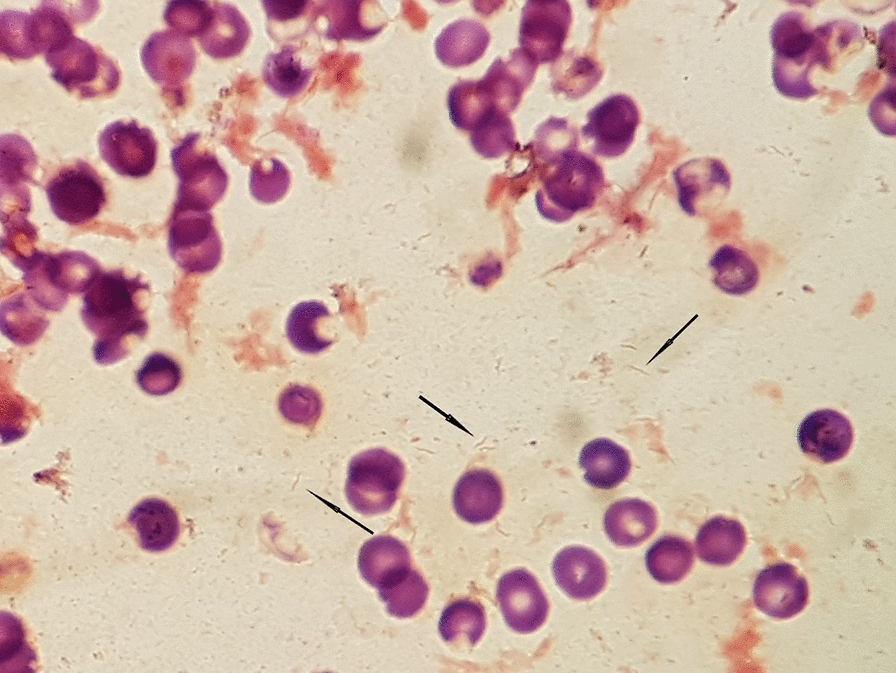
Gram stain of a positive aerobic blood culture bottle. Black arrows indicate pale, thin Gram-negative spiral-shaped rods

**Fig. 3:**
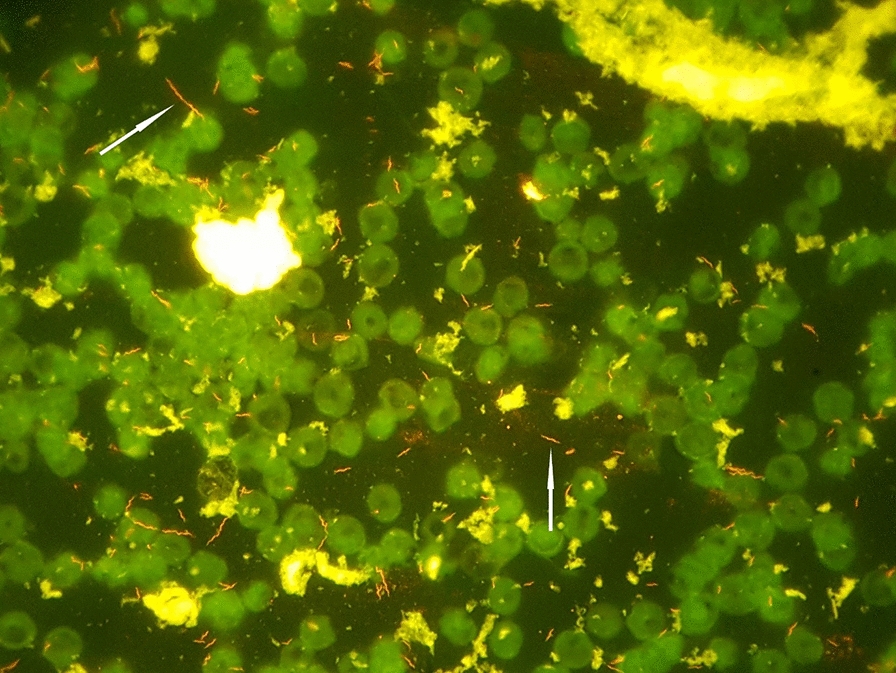
Acridine orange stain of a positive aerobic blood culture bottle. White arrows indicate spiral-shaped rods

**Fig. 4:**
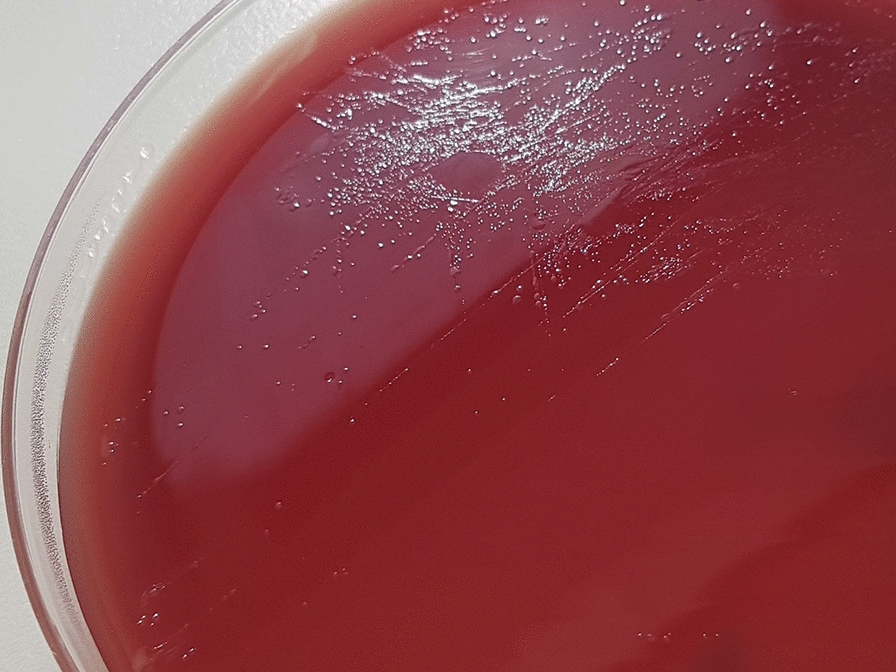
Brucella agar plate after 3 days of incubation at 37 °C in microaerophilic conditions showing translucent round or irregularly shaped colonies

**Fig. 5:**
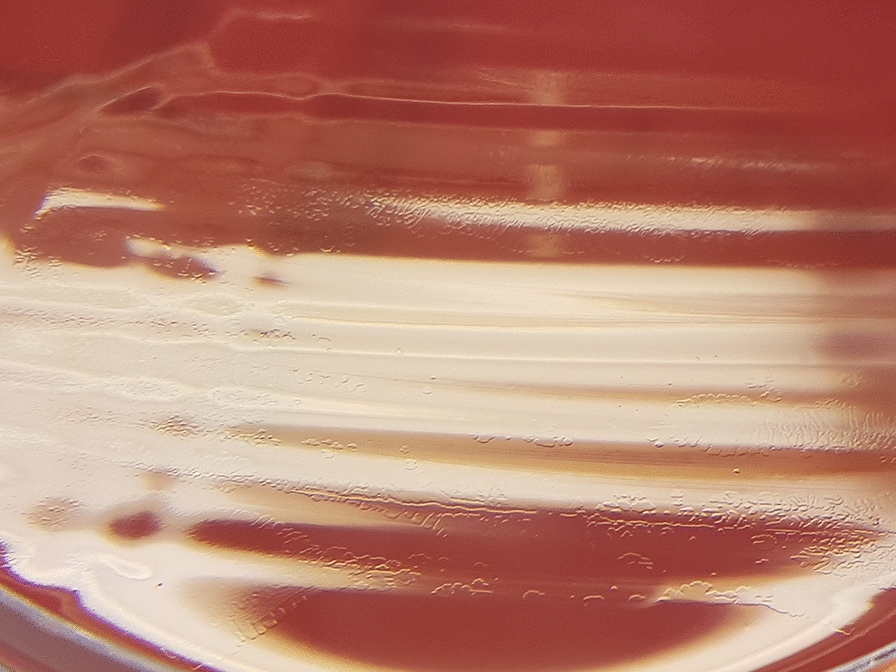
Colombia sheep agar plate after 6 days of incubation at 37 °C in microaerophilic conditions showing swarming phenomenon

The same day, blood samples were collected for culture. Blood was drawn in 4 blood culture bottles (two aerobic, two anaerobic). After 5 days of incubation (BACT/ALERT VIRTUO, BioMerieux), both aerobic bottles flagged positive. Gram stain showed the presence of Gram-negative spiral rods (Fig. 2). As it was difficult to establish the exact morphology of rods in Gram stain, another smear stained with Acridine Orange was performed (Fig. 3). Direct identification from bottles using Sepsityper kit and MALDI-TOF MS (Bruker Daltonik) did not yield any result. Suspecting an organism of fastidious growth after performing Gram stain, bottles were then subcultured on a set of several different media, including blood agar, Colombia agar with 5% horse blood (BioMerieux), Colombia agar with 5% sheep blood (BioMerieux) and Brucella agar (Oxoid), all under different temperature (37 and 42 °C) and atmosphere conditions, including aerobic with 5% CO_2_ and microaerophilic atmosphere. After 48 h, the only media with visible growth, though extremely scant, was Colombia sheep agar incubated in microaerophilic atmosphere at 37 °C. After 72-96 h, growth was observed on all media incubated in microaerophilic conditions, at 37 °C as well as 42 °C, although the growth was better at 37 °C. Shiny translucent colonies of round or irregular shape were best observed on Brucella agar (Fig. 4). After 6 days of incubation, a phenomenon of swarming was observed on Colombia sheep agar, incubated in microaerophilic conditions at 37 °C (Fig. 5). The result of identification by MALDI-TOF MS from culture was *H. canis.* However, score was insufficient for reliable result (1.88).

Additionally, 16 S rRNA sequence analysis of isolate was performed. DNA from pure culture was extracted with Chelex 100 Resin (BioRad). Amplification of the full (1,5 kb) 16 S rRNA gene was performed as described previously by Bianciotto et al. [23]. Amplified 16 S rDNA were sequenced on 3500 Genetic Analyzer using the BigDye Terminator Kit (Applied Biosystems). The forward and reverse strands were aligned using BioNumerics v7.5 (Applied Maths) and the 16 S rDNA sequence was then compared with entries in MicrobeNet (https://www.cdc.gov/microbenet/index.html).

The 16 S rRNA sequence analysis confirmed identification of *H. canis* previously obtained with MALDI-TOF MS. Antimicrobial susceptibility testing, performed with gradient E-test MIC method (ETEST - BioMerieux), showed low minimum inhibitory concentrations (MICs) to amoxicillin-clavulanate, cefotaxime, ceftriaxone, meropenem, and gentamicin (Table 2).

**Table 2:** Results of antimicrobial susceptibility testing of *H. canis* isolated strain

Antimicrobial substance	Amoxicillin-clavulanate	Cefotaxime	Ceftriaxone	Meropenem	Gentamicin
MIC [µg/mL]	0.016	0.125	0.25	0.25	0.064

*MIC* minimum inhibitory concentration

On the 9th day, after *H. canis* infection identification, combined antibiotic regimen with ceftriaxone 2 g daily intravenously and doxycycline 100 mg twice daily perorally for 14 days was initiated. The antibiotic regimen was chosen according to previous case reports [5, 13]. Five days after combined antibiotic treatment initiation, markers of inflammation decreased (leukocyte blood count 5.31 × 10^9^/L, C-reactive protein 11 mg/L).

In between, due to anaemia and increased markers of inflammation, other foci of inflammation or malignancy were investigated [24]. However, none were found. Ultrasound of the abdomen revealed gallstones. Esophagogastroduodenoscopy showed chronic superficial gastritis. Ultrasound of the heart showed no significant abnormalities. Additionally, serology testing for *Borrelia burgdorferi*, *Leptospira spp*., and stool culture (*Salmonella spp., Shigella spp., Campylobacter spp., Yersinia spp., E. coli O157, Shiga toxin-producing E. coli*) were negative.

Before discharge, blood samples for control blood cultures were collected. All were sterile. Furthermore, primary sterile samples were tested for 16 S rRNA gene sequencing. All samples were negative for 16 S rRNA gene of *Helicobacter* species at discharge.

## Discussion and conclusions

In this case report, we described a female patient with bacteraemia caused by *H. canis* and RA treated with tofacitinib in combination therapy. Infection occurred 8 months after treatment initiation. According to previous case reports (Table 1), clinical presentation of *H. canis* bacteraemia is diverse. Three case reports described multifocal cellulitis with or without fever episodes [5, 8, 9]. Four others described intermittent and/or recurrent fever only [10, 11, 13, 14]. One case in an infant ended with a cardiorespiratory arrest [15]. Our case described a patient with lumboischialgia that resulted from multilevel degenerative lumbar spinal stenosis. Apart from increased markers of inflammation and clinical presentation of spinal stenosis, our patient had asymptomatic *H. canis* bacteraemia. We present the first case describing laboratory findings of *H. canis* bacteraemia only. However, our patient received chronic non-steroidal anti-inflammatory drug and paracetamol therapy which could mask febrile episodes frequently presented in other patients [25].

Important risk factor for *H. canis* bacteraemia might be an incompetent immune system. Majority of previous patients with bacteraemia were immunocompromised. One case described bacteraemia in a patient with X-linked hypogammaglobulinemia [8]. Another case described a patient with common variable immune deficiency, sarcoidosis, and splenectomy [13]. Furthermore, two case reports described patients receiving immunosuppressive drugs. The first case described a patient with B cell lymphoma treated with R-CHOP chemotherapy [11]. The second case described a kidney transplant patient treated with tacrolimus, mycophenolate mofetil, and prednisone [14]. Our patient had a combination of both risk factors.

On the one hand, she had RTX-induced hypogammaglobulinemia. On the other hand, she was treated with tofacitinib, MTX, and a low methylprednisolone dose. A recent large cohort study found that patients with RA, receiving tofacitinib in combination with MTX, have had 3.0% risk of severe infections, among them, 0.2% risk for tuberculosis (TBC), 4.1% risk for herpes zoster, and 0.6% risk for opportunistic infections excluding TBC [26]. The risk of serious infections was higher in patients older than 65 years [26].

Another factor contributing to a low number of *H. canis* case reports might be challenging and easily overlooked laboratory recognition. *H. canis* can grow in standard aerobic blood cultures, however, in general, micro-aerophilic atmosphere conditions (37 or 42 °C) are needed for growth [5]. Furthermore, it is relatively inert biochemically - catalase, urease, and nitrate negative [13]. Until recently, identifying fastidious organisms like NHPS was challenging in most clinical laboratory settings. Because of their low biochemical reactivities, differentiation methods relying on isolates’ biochemical characteristics were unsuccessful. With the development and implementation of newer identification methods like MALDI-TOF MS and molecular methods like 16 S rRNA gene sequence analysis, accurate identification of these fastidious microorganisms became readily available [5, 13, 27].

Currently, no recommendations for treatment of *H. canis* bacteraemia are available. Previous case reports described different treatment regimens. Some patients were treated with amoxicillin-clavulanate [5] or ceftriaxone [9], whereas others were treated with a combination of ceftriaxone and doxycycline [13] or a combination of cefuroxime and ciprofloxacin [14]. A minimum of 2 weeks and a maximum of 8 weeks of therapy were described. We successfully treated our patient with a combination of ceftriaxone and doxycycline in a duration of 14 days. To point out, this is the third case where results of *in vitro* antimicrobial susceptibility testing are presented. Like our results, Leeman et al. found susceptibility of *H. canis* to amoxicillin, amoxicillin-clavulanate, ceftriaxone, piperacillin-tazobactam, imipenem, metronidazole, and clindamycin [9]. Other study found susceptibility to nalidixic acid only [10]. Clear recommendations for optimal antibiotic treatment regimen of *H. canis* infections cannot be made. However, this is the fifth case where either a third-generation cephalosporin or doxycycline was used and resulted in complete remission of *H. canis* infection [8, 9, 13, 14].

In conclusion, *H. canis* infections are rare and difficult to diagnose. Clinicians should recognise risk for infection in patients with recent exposure to dogs, cats or sheep while not improving with standard therapy. Risk further increases with underlying comorbidities, especially, autoimmune diseases, immunodeficiency disorders, or use of immunosuppressive agents. A minimum of 2 weeks of therapy is suggested [13].

## Data Availability

The datasets are available from the corresponding author on reasonable request.

## Abbreviations

CVID: Common variable immune deficiency
*H. canis*: *Helicobacter canis*
*H. pylori*: *Helicobacter pylori*
IgG: Immunoglobulin G
IgM: Immunoglobulin M
JAKi: Janus kinase inhibitors
MALDI-TOF MS: Matrix-assisted laser desorption/ionisation-time of flight mass spectrometry
MTX: Methotrexate
NHPS: Non-*Helicobacter pylori* species
RA: Rheumatoid arthritis
R-CHOP: Rituximab -cyclophosphamide, hydroxydaunorubicin, oncovin, prednisone
rRNA: Ribosomal ribonucleic acid
RTX: Rituximab
MIC: Minimum inhibitory concentration
